# The interaction of carcinogenic metals with tissues and body fluids. Cobalt and horse serum.

**DOI:** 10.1038/bjc.1969.23

**Published:** 1969-03

**Authors:** J. C. Heath, M. Webb, M. Caffrey

## Abstract

**Images:**


### THE INTERACTION OF CARCINOGENIC METALS WITH TISSUES AND BODY FLUIDS. COBALT AND HORSE SERUM

J. C. HEATH, M. WEBB AND MAEVE CAFFREY

*From the Strangeways Research Laboratory, Cambridge*

Received for publication September 30, 1968

It has been shown in this laboratory that malignant tumours, mostly rhabdomyosarcomata, are readily induced in rat skeletal muscle by implants of powdered metallic cobalt (Heath, 1956).

In histogenetic studies of these cobalt-induced tumours (Heath, 1960) the stages of initial muscle damage followed by regeneration and attempts at repair are clearly defined, and the emergence of abnormal and later malignant myoblasts, can readily be followed. It is reasonable to suppose that the free myoblasts taking part in the regeneration process are subject to a concentration gradient of dissolved cobalt. Initially, it was assumed that this metal was probably in the ionic form, since it was observed that the metal slowly disappeared from the injection site by some process other than phagocytosis.

As cells in culture usually respond to an experimental agent in much the same way as cells *in vivo*, experiments were made in which cultures of rat myoblasts and fibroblasts were treated with cobalt chloride in various concentrations, in the hope of reproducing *in vitro* the premalignant changes observed *in vivo*. The results were disappointing, however. At concentrations above a certain level, the cells were killed; at sublethal concentrations they appeared unaffected, and showed no cytological abnormalities like those in the early muscle tumours.

From these negative results, together with additional evidence (unpublished experiments) that, at least in suspensions of mouse ascites cells and rat dermal fibroblasts, ionic cobalt was bound mainly at the cell surface and was not incorporated intracellularly, it seemed unlikely that ionic cobalt was the carcinogenic agent *in vivo* as originally supposed. Since a period of several weeks elapses between the implantation of the metal and the first appearance of the premalignant cytological changes, it seemed possible that the metallic cobalt might slowly interact with a tissue component and that the complex thus produced might be the carcinogenic factor. It was thought that such a complex might be more easily taken up by the cell than ionic $Co^{2+}$; its subsequent breakdown by intracellular enzymes might then liberate cobalt into susceptible regions and thus initiate the malignant changes.

The results described in this paper show that powdered metallic cobalt dissolves slowly when incubated aseptically with horse serum, to yield a solution of characteristic colour in which much of the dissolved metal is bound to protein. Not only is the cobalt in this form less toxic to rat myoblasts in culture than the equivalent concentration of $CoCl_2$, but also it produces in actively growing cultures (containing many dividing cells) cytological alterations similar to those seen in the premalignant myoblasts *in vivo*.

### MATERIALS AND METHODS

### *Serum and serum fractions*

Sterile fresh horse serum was obtained in 200 ml. lots at weekly intervals from the School of Veterinary Medicine, University of Cambridge. This serum was stored at 0° C. until 4 or 5 lots had accumulated when these were mixed and refiltered under pressure through a Millipore filter grade 0·45 $\mu$. This pooled serum was again stored at 0° C. and then used as required for both culture and for incubation at 37° C. with powdered metallic cobalt.

Equine albumin (Fraction V, B-grade), globulins (Fraction III, B-grade) and bovine transferrin (B-grade) were obtained from Calbiochem (Los Angeles, California, U.S.A.).

*Chemicals.*—Spectrographically standardised cobalt metal powder was purchased from Johnson, Matthey and Company Limited (Hatton Garden, London, E.C.1). Penicillamine was generously given by Dista Products, Speke, Liverpool. Cobalt glycylglycine was prepared by the method of Gilbert, Otey and Price (1951). Copper complexes of the amino acid components of a commercial casein hydrolysate (" casamino acids ", Difco Laboratories Inc., Detroit, Mich., U.S.A.) were made by the method of Tommel, Vliegenthart, Penders and Arens (1968). Cobalt complexes were made similarly from freshly prepared basic cobalt carbonate. All other chemicals were of Analar, or equivalent grade.

*Enzymes.*—An acetone-powder of a combined lysosomal and mitochondrial fraction from chicken liver was provided by Dr. A. J. Barrett. This contained 1·1 units of lysosomal proteinase/mg. when assayed by the method of Barrett (1967). A crude preparation of the alkaline proteinase of rat skeletal muscle was made by the method of Koszalka and Miller (1960); the activity of this enzyme was determined by the formation of products that remained soluble after the addition of an equal volume of 5% (w/v) trichloroacetic acid (TCA). The reaction was followed spectrophotometrically by the increase of absorbance at 280 m$\mu$. With 4% (w/v) serum albumin as substrate in 0·24 M tris-HCl buffer pH 9·0 (4·0 ml.) an increase in absorbance of 0·14 was produced by 1 ml. of the enzyme solution in 20 min. at 37° C.

### *Incubation of horse serum with metallic cobalt and $CoCl_2$*

Sterile horse serum (50 ml.) was incubated aseptically at 37° C., with powdered metallic cobalt (15 mg.) in a Pyrex glass stoppered 100 ml. flask which was agitated gently at daily intervals. Aerobic and anaerobic incubations were done with air and nitrogen respectively as the gas phase. In some experiments, the cobalt metal was replaced by an equivalent amount of $CoCl_2 6H_2O$.

### *Analytical and preparative procedures*

Spectrophotometric measurements were made in either a Unicam SP 500 or SP 700 spectrophotometer. Aerobic preparations of the incubated sera were clarified by centrifugation before measurement. Anaerobically incubated mixtures were transferred *via* a cotton filter pad from the reaction vessel to the evacuated spectrophotometer cell under a positive pressure of nitrogen.

Dialysis was done in Visking tubing, which was pretreated with 1 mM ethylenediamine tetra-acetic acid (EDTA) in 0·1 M tris HCl buffer, pH 7·4, and then washed repeatedly in glass-distilled water.

Cobalt was determined by atomic absorption as previously described (Heath and Webb, 1967). Protein solutions were digested before analysis with either concentrated $HNO_3$ or $H_2SO_4$, the blanks being treated similarly.

Gel electrophoresis was done by the method of Davis (1964), the samples being dissolved initially in 0·2 M sucrose.

Protein was estimated as described by Lowry *et al.* (1951).

*Column chromatography*

*Fractionation of serum proteins.*—This was done on columns of Sephadex G-150 (particle size 40–120 $\mu$. Pharmacia Uppsala, Sweden) which were packed and run with 1% (w/v) NaCl as eluent as described by Flodin (1962). The fractions were analysed for protein by measurement of $E_{280}$ ($E_{280} \equiv 1{\cdot}0$ for 1 mg. protein/ml.) and for cobalt by atomic absorption. After separation, the appropriate fractions from the columns were combined and concentrated in LKB ultrafilters. For culture work these solutions were diluted to contain 0·8% (w/v) NaCl, supplemented with the other components of Tyrode solution and sterilized by filtration through Millipore membranes.

*Separation of cobalt complexes on Chelex.*—Protein-bound cobalt in cobalt-serum was not retarded on Chelex analytical grade chelating resin (100–200 mesh; Bio-Rad Laboratories, Richmond, California, U.S.A.) in the sodium-hydrogen-form, and thus was separated from ionic $Co^{2+}$ which was retained at the top of the column. The anionic cobalt glycylglycine complex was also eluted from Chelex columns with the solvent front (water or 1% (w/v) NaCl), whereas both the copper and cobalt complexes of the mixed amino acids in a casein hydrolysate were bound firmly. Cations retained by the resin were eluted with 1·0 *N* HCl.

*Enzymic hydrolysis of whole and fractionated cobalt-serum*

A solution of the acetone-dried powder of chicken liver lysosomal proteinase (5·5 mg.) in 1·0 M acetate buffer, pH 4·0 (1·0 ml.) was added to cobalt serum, or equivalent solution (10 ml.), previously dialysed against 0·15 M NaCl. The mixture was sterilized by filtration through a Millipore membrane, incubated at 45° C. for 18 hours and then transferred aseptically to a sterile dialysis tube. After dialysis for 24 hours against 3 changes each of 100 ml. of sterile glass-distilled water, the combined diffusates were frozen dried. A solution of this residue in water (5 ml.) was applied to a Chelex column (10 cm. × 2·2 cm. diam.) with water as the initial eluent. $Co^{2+}$ bound to the resin was eluted with 0·1 N HCl.

*Photo-oxidation of cobalt serum*

This was done essentially as described by Wood and Bannister (1967). Illuminated mixtures of dialysed cobalt-serum, or of control serum (22·5 ml.), 0·2 M phosphate buffer, pH 8·5 (11·25 ml.) and methylene blue (0·02% (w/v)) in 0·1 M NaCl (11·25 ml.) were shaken in a water-bath at 37° C. Mixtures (2·0 ml.) of the same composition were incubated in Warburg flasks under the same conditions for the measurement of oxygen consumption. After 2 hours the solutions were removed from the bath and dialysed against 0·1 M NaCl (total vol. 300 ml.) for 18 hours in the dark. The combined diffusates were concentrated by rotary-evaporation and passed through a Chelex column. $Co^{2+}$ retained by the resin was eluted with 0·1 N HCl.

*Mouse ascites tumour cells.*—(Strain BALB/c) were maintained by passage in $C_3H$ mice and, for experimental purposes, were collected 5 days after intraperitoneal injection. The cells were washed four times in phosphate-buffered Krebs Ringer saline, heparin (10 $\mu$g./ml.) being included in the initial wash, and then suspended in the same saline at 1·0–1·5 $\times$ $10^7$ cells/ml.

*Rat liver mitochondria* were isolated by Schneider's (1948) method. Suspensions (in 0·25 M sucrose and 5 mM tris-HCl buffer, pH 7·4) were filtered through cotton gauze before being used in the manometric experiments.

*Manometric methods.*—Oxygen consumption by ascites tumour cells (5·0 $\times$ $10^6$ cells/flask) and rat liver mitochondria (1 mg. protein N/ml.) was measured in the conventional Warburg apparatus. Mitochondria were incubated in the medium of Dingle *et al.* (1962) with 5 mM pyruvate (in 0·05 mM funarate) as substrate.

*Culture of embryonic rat myoblasts.*—Skeletal muscle was dissected from the backs of 14–15 day rat embryos, washed in $Ca^{2+}$ and $Mg^{2+}$—free Tyrode and dissociated in 1% (w/v) trypsin (Difco Bactotrypsin 1 : 250) with 0·002% (w/v) EDTA. After the addition of an equal volume of horse serum to inhibit the trypsin, the cells were centrifuged at low speed and washed with culture medium. The latter always contained 8 vol. Tyrode solution, 3 vol. horse serum and 1 vol. 50% chick embryo extract (prepared by extraction of minced 12 day embryos with an equal volume of Tyrode solution).

The cells (myoblasts) were plated out in horizontal 8 oz. baby-feeding bottles, each of which contained a 10 $\times$ 38 mm. coverslip and medium (4 ml.) which was partially changed every 2nd or 3rd day. All cultures were trypsinised each week and subcultures established.

When the myoblasts were treated with cobalt-serum, the latter was diluted with horse serum such that an effective but non-lethal level of cobalt was attained. This level was between 2 and 4 $\mu$g. $Co^{2+}$/ml. and usually about 3·3 $\mu$g. $Co^{2+}$/ml. Once treated with cobalt serum the cells were kept always in this medium.

Coverslips were removed from each culture prior to trypsinisation, rinsed quickly in Tyrode solution at 37° C., and fixed in absolute methanol. The preparations were then stained in methyl-green-pyronin and (duplicates) in May-Grünwald-Giemsa.

### RESULTS

### *Dissolution of cobalt metal in horse serum*

Powdered metallic cobalt dissolved slowly when incubated aerobically at 37° C. with either buffered, neutral solutions of simple chelating agents such as penicillamine and EDTA (Fig. 1), or with horse serum (Fig. 2). Solution of the metal in horse serum was inhibited by the absence of oxygen, and was increased by the presence of an additional chelating agent (e.g. penicillamine, Fig. 2).

With different samples of horse serum approximately the same amounts of metal (208–222 $\mu$g. $Co^{2+}$/ml.) were dissolved in 28 days at 37° C. (Corresponding values for rat and calf sera were 214 and 148 $\mu$g. $Co^{2+}$/ml. respectively.) Usually, incubation was terminated after this time, but in metal serum mixtures that were kept for longer periods at 37° C., or even 0° C., the contents of dissolved metal continued to increase slowly. Metal granules that remained undissolved after 1 month had at least a surface film of sulphide and evolved $H_2S$ on treatment with 5 N HCl.

Probably through denaturation and polymerisation of certain protein components (e.g. Pederson, 1962; Findlayson, 1965) separation of some insoluble material, as a surface skin and as a granular precipitate, occurred in serum alone after incubation for several days. In cobalt serum mixtures these insoluble fractions appeared larger, possibly because of additional protein precipitation by the dissolving cobalt (cf. Aoki *et al.*, 1967). After elimination from cobalt serum of the insoluble components which bound only small amounts (about 1·5–1·7 $\mu$g. $Co^{2+}$/ml. serum) of the dissolved $Co^{2+}$, decreases in the contents of both $\beta$- and $\gamma$-globulin fractions were detected by gel electrophoresis.

FIG. 1.—Solution of metallic cobalt in Tyrode's solution alone (□—□), with the addition of penicillamine (2 mg./ml. ●—●) and with neutralised (pH 7·4) EDTA (5 mg./ml. ○—○).

The $Co^{2+}$ concentration of cobalt serum was reduced by 30 to 50% on exhaustive dialysis against 4 changes, each of 10 times the serum volume, of 0·154 M NaCl in a rocking dialyser. Somewhat greater amounts of diffusible $Co^{2+}$ were removed by dialysis against 0·154 M NaCl than against water. With one sample of cobalt-serum, on which comparative measurements were made, 50% and 30% of the total $Co^{2+}$ was removed by dialysis against saline and water, respectively. This difference, however, is not unequivocal evidence that part of the dissolved $Co^{2+}$ is ionically bound at free carboxyl groups of the serum proteins and is displaced by $Na^{+}$ ions, since the physical properties of the system alter during dialysis against water through the precipitation of globulins. Slight precipitates were formed during dialysis against 0·154 M NaCl; these contained $Co^{2+}$ equivalent to about 1·3 μg. $Co^{2+}$/ml. serum.

Although more of cobalt was dissolved by serum-penicillamine mixtures than by serum alone (Fig. 2), this excess $Co^{2+}$ was bound by the penicillamine. On dialysis of the former solution (340 μg. $Co^{2+}$/ml.) the dark brown $Co^{2+}$-penicillamine chelate was removed and the content of $Co^{2+}$ in the dialysis residue (150 μg. $Co^{2+}$/ml.) approached that of the corresponding fraction from the cobalt-serum (110 μg. $Co^{2+}$/ml.).

FIG. 2.—Solution of metallic cobalt in sterile horse serum at 37° C. with either air (●—●), or nitrogen (□—□) as the gas phase, and in sterile serum with penicillamine (2 mg./ml. ○—○) in air.

Loss of diffusible $Co^{2+}$ by dialysis against 0·154 M NaCl was accompanied by a decrease in pH from pH 7·8–7·9 to pH 7·0–7·2. No further significant change in $Co^{2+}$ content occurred on re-dialysis of the serum against isotonic buffers of pH 6 to pH 9, but in more acid solutions (pH 3 and 4) additional amounts (26% and 14% respectively) of the cation were removed. Also further dialysis against a solution of 10 mM EDTA in a mixture of 10 mM tris buffer, pH 7·5, and 0·14 M NaCl, reduced the content of protein-bound $Co^{2+}$ by 50–60%. The residual non-diffusible $Co^{2+}$ ions were not removed from the serum proteins by Chelex resin, but were extracted almost quantitatively by TCA (final concentration of 5% (w/v)) at

0° C. At 60° C. less $Co^{2+}$ (about 35%) was liberated by 5% (w/v) TCA than at 0° C.

As the cobalt metal dissolved, the colour of the serum changed to a dark reddish brown. A scan of the difference spectrum (i.e. with incubated, normal serum in the reference cuvette) established the production of absorption maxima at 370–390 mμ and 520–530 mμ. With 0·154 M NaCl or $H_2O$ in the reference cell the former peak was largely obscured by the intense absorption due to protein, whilst the latter was reduced to a shoulder. Much of the absorption in the cobalt-serum was due to coloured complexes of $Co^{2+}$ with small molecular components, which

Fig. 3.—Solution of metallic cobalt (continuous lines) in sterile whole serum and in dialysed serum at 37° C. with air as the gas phase. The lower curves (dashed lines) show the amounts of $Co^{2+}$ (right hand axis) that precipitated as insoluble salts at different times during the incubation, ●, whole serum; ○, dialysed serum.

were removed by dialysis against either 0·154 M NaCl or water. These diffusates contained little or no ionic $Co^{2+}$ which was retained on Chelex resin, and their concentrated solutions, in common with solutions of a number of $Co^{2+}$-peptide complexes (e.g. glycylglycine, glycyl-D-alanine and sarcosylglycine, Smith, 1948*a*, 1948*b*) had absorption maxima at 520 mμ. After exhaustive dialysis against 0·154 M NaCl, however, a light reddish-brown colour ($\lambda_{max.}$ 510–520 mμ) persisted in the cobalt sera.

Although a significant part of the soluble cobalt of cobalt serum was complexed with small molecules, the powdered metal was dissolved equally effectively by sterile serum from which the diffusible components had been eliminated by dialysis (Fig. 3). In predialysed serum, however, additional amounts of $Co^{2+}$ separated from solution after incubation for several days as a pink, acid-soluble precipitate probably $Co(OH)_2$ (Fig. 3).

Horse serum incubated in air with $CoCl_2$ (200 $\mu$g. $Co^{2+}$/ml.) showed changes in absorption which were similar to those produced on incubation with the metal. Under nitrogen, little change in absorbance occurred. Aerobically, absorption maxima at 370–390 m$\mu$ and at 520 m$\mu$ were detected after 48 hours at 37° C. and increased steadily in intensity during the first 12 days of incubation. Dialysis of these preparations after this time against 0·154 M NaCl yielded non-dialysable and diffusible fractions, the absorption spectra of which were qualitatively similar to those of the corresponding fractions from serum that had been incubated with cobalt metal. It appears, therefore, that the products formed on incubation of horse serum with cobalt metal and $Co^{2+}$ ions are chemically similar. Some difference in biological activity, however, suggests that these products may not be identical; hence, the term $Co^{2+}$-serum is used subsequently in the text to distinguish a preparation that was obtained by incubation of horse serum with $CoCl_2$ from that (cobalt serum) produced by the dissolution of the metal.

*Photo-oxidation of cobalt serum*

Photo-oxidation of proteins in the presence of methylene blue and oxygen causes the destruction of histidyl residues at rates that are much greater than those with other photo-sensitive amino-acids (Weil and Buchert, 1951; Weil and Seibler, 1953). With the metalloprotein, haemocyanin, the $Cu^{2+}$ content is decreased on photo-oxidation, from which it is inferred that $Cu^{2+}$-ions are bound at histidyl residues (Wood and Bannister, 1967). Similar experiments with cobalt-serum showed that the rate of photo-oxidation of this complex was about 25–30% less than that of serum alone. With both cobalt-serum and control serum the rate of oxygen uptake decreased significantly after the consumption of about 18 $\mu$ atoms oxygen/ml. serum. From the products of the photo-oxidation of the cobalt serum only 13–14% of the protein-bound $Co^{2+}$ was separated by dialysis and was recovered on Chelex resin. It seems unlikely, therefore, that chelation of histidine residues can account for more than a small fraction of the $Co^{2+}$ bound in cobalt-serum.

*Fractionation of cobalt-serum*

Cobalt-serum (or $Co^{2+}$-serum) was separated on Sephadex G-150 into three protein fractions (Fig. 4). As expected from the work of Flodin (1962), gel electrophoresis of these protein fractions, after concentration by either freeze-drying or ultrafiltration, showed that the globulins were contained in Peaks I and II, and albumin, together with minor components that occurred at positions intermediate between those of the reference markers, albumin and transferrin, in Peak III. No consistent or significant differences were observed in the electrophoretic patterns of Peak III from cobalt-serum and of the corresponding fraction from normal serum (see below) when these were run at approximately the same protein concentration.

Each protein peak in the elution pattern of the components of the cobalt-serum coincided with a maximum in $Co^{2+}$-content (Fig. 4). The highest concentration of $Co^{2+}$ was associated with the albumin fraction (peak III), concentrated solutions of which had high biological activity in the tissue culture system, and were reddish-brown in colour ($\lambda_{max.}$ = 520–530 m$\mu$).

Incubated preparations of normal serum were also resolved on Sephadex G-150 into three fractions, the protein concentration of the second (in contrast to that from cobalt-serum, Fig. 4) usually being greater than the first or third. None of the fractions from normal serum had any significant effect upon the growth and morphology of rat myoblasts *in vitro*.

FIG. 4.—Fractionation of cobalt-serum by gel-filtration on Sephadex G-150. - - - ● - - -, $Co^{2+}$ concentration (μg./ml.); —○—, protein concentration. The cobalt contents (μg./mg. protein) at the 3 maxima were (I), 0·79, (II), 0·59 and (III) 2·48 respectively.

*Liberation of $Co^{2+}$ from the components of cobalt serum*

As with the original cobalt-serum, $Co^{2+}$ ions were bound firmly to the protein components of fractions I, II and III (see above) and were not removed by Chelex resin, or extracted completely by 5% (w/v) TCA at 60° C.

Hydrolysis of dialysed, but unfractionated, cobalt-serum with preparations of proteolytic enzymes from both chicken liver lysosomes and rat skeletal muscle was accompanied by the liberation of $Co^{2+}$ in a form that was retained by Chelex. Thus in a typical experiment, 58·5% of the bound $Co^{2+}$ of cobalt-serum (90·2 μg. $Co^{2+}$/ml.) was rendered diffusible on digestion with the chicken liver lysosomal proteinase (see " Materials and Methods " section). Of this diffusible $Co^{2+}$, 23% was present in peptide complexes that were not retained by Chelex resin. The remainder (77%) was eluted from the column with 1 N HCl and appeared to be free from amino acids detectable with ninhydrin.

The three protein fractions that were separated from cobalt-serum on Sephadex G-150 (Fig. 4) differed in susceptibility to hydrolysis by the lysosomal proteinase, more diffusible $Co^{2+}$ being liberated from fraction III than from either fraction I or fraction II (Table I).

TABLE I.—*Liberation of Diffusible $Co^{2+}$ on Hydrolysis of Fractions of Cobalt-Serum by the Lysosomal Protein of Chicken Liver*

In the 2 experiments recorded the protein fractions were adjusted to contain approximately equal amounts of (*a*) protein (6·8 mg./ml.) and (*b*) $Co^{2+}$ (11 μg./ml.), and were incubated with the enzyme at pH 4·0 and 45° C. as described in the " Materials and Methods " section. Diffusible $Co^{2+}$ was separated by dialysis against three changes, each of 10 vol., of glass-distilled water.

| Fraction of cobalt-serum | Diffusible $Co^{2+}$ (% of total) recovered after incubation with lysosomal proteinase at constant concentrations of | |
|---|---|---|
| | Protein | $Co^{2+}$ |
| I | 52·8 | 42·6 |
| II | 58·1 | 55·6 |
| III | 63·9 | 76·2 |

*Effect of cobalt-serum on cultures of rat myoblasts*

Cobalt in cobalt-serum was much less toxic to cultures of rat myoblasts than an equivalent concentration of $CoCl_2$. In actively growing cultures, containing many dividing cells, the cobalt serum produced cytological changes similar to those seen in the premalignant myoblasts *in vivo*. These changes included enlarged hyperchromatic nucleoli, chromocentres and nuclei (Fig. 7, cf. Fig. 5 and 6), and increased pyronin-staining of the cytoplasm, indicating a raised content of ribonucleic acid. After 2–3 weeks cultivation in the cobalt-serum at the concentration used, these effects became more pronounced with 80–95% of all interphase cells showing the typical changes whilst the rate of mitosis was depressed. The cells were still alive, however, and resumed growth when returned to normal medium. Control cultures maintained in serum incubated without cobalt were unaffected.

*Biological activities of cobalt-serum fractions*

The fractions that were separated from cobalt-serum by gel-filtration on Sephadex (i.e. fractions I, II and III; Fig. 4) were incorporated into the culture medium as described in the " Materials and Methods " section to give the same cobalt concentration as that known to be effective with cobalt-serum, and tested against rat myoblasts in culture. Since the $Co^{2+}$ contents of these fractions were very different (e.g. fraction I, 1·1; fraction II, 0·5 and fraction III, 1·5 μg. $Co^{2+}$/mg. protein), this procedure introduced appreciable and uncontrolled variations in the

---

EXPLANATION OF PLATE

FIG. 5.—Control culture of rat myoblasts. In this figure, as in Fig. 6 and 7, the culture was fixed in methanol and stained with methyl-green and pyronin. × 1200.

FIG. 6.—Rat myoblasts in culture treated with cobalt chloride to give a concentration of 2 μg. $Co^{2+}$/ml. All cells are poisoned.

FIG. 7.—Rat myoblasts in culture treated with cobalt-serum to give a concentration of 2 μg. cobalt/ml. The photograph shows the typical non-lethal changes in interphase cells.

5 6

7

Heath, Webb and Caffrey.

protein concentration of the culture media. With the limitations imposed by these variations in the experimental system, fraction III was the most active of the three in the induction of the above-mentioned cytological changes, whilst fraction I appeared to be rather toxic.

*Effect of cobalt-serum*

(1) *On respiration of ascites tumour cells.*—The cobalt serum complex (1 ml. ≡ 78 $\mu$g. $Co^{2+}$) was not inhibitory to the rate of oxygen consumption (4·7 $\mu$l. $O_2$/hr./

FIG. 8.—Inhibition of pyruvate oxidation in suspensions of rat liver mitochondria by $Co^{2+}$, cobalt-serum and $Co^{2+}$ in the presence of whole serum. Additions to the experimental system (see " Materials and Methods " section) were as follows: ●, none; ■, $Co^{2+}$ (as $CoCl_2$); □, cobalt-serum; ○, horse serum; ▲, horse serum + $Co^{2+}$. The concentration of ionic or bound $Co^{2+}$ in (*a*) was 40 $\mu$g./ml. and in (*b*) 60 $\mu$g./ml.

$10^6$ cells) by ascites tumour cells over a four-hour period and, in some measurements, appeared slightly stimulatory (up to 10%) to respiration. Ionic $Co^{2+}$ (78 $\mu$g.), in contrast, inhibited oxygen uptake by 38–40%. This inhibition was reduced to about 11% in the presence of horse serum (1 ml.).

(2) *On mitochondrial pyruvate oxidation.*—Inhibition of pyruvate oxidation in suspensions of rat liver mitochondria by cobalt-serum was less than, but similar to, that produced by an equivalent concentration of ionic $Co^{2+}$ (Fig. 8). The activity of the latter, however, was reduced to less than that of the former by the addition of sufficient whole serum to give the same protein concentration in both systems.

Presumably this was due to binding of $Co^{2+}$ by small molecular ligands present, in addition to protein, in the whole serum.

### DISCUSSION

Although investigations on the mechanism of solution of metallic cobalt in sterile serum at neutral pH are still in progress, the requirement for oxygen suggests that the process is coupled with a cycle of oxidation and reduction of certain organic molecules, which might involve the formation of free radicals. As the metal also dissolves in buffered solutions of chelating agents of low molecular weight, it is likely that studies with these compounds will provide useful experimental models for the more complicated serum system. As has been discussed by Todd (1965 and personal communication) both changes in redox potential and the formation of free radicals in the neighbourhood of the implanted metal *in vivo* might be significant in the induction of malignancy.

About 50% of the cobalt that dissolves in serum during 1 month at 37° C., is bound to protein and is not removed by dialysis against isotonic saline. Fractionation experiments (Fig. 4) show that this bound cation is not associated with a specific metal-binding protein, but is distributed amongst the globulin and albumin components of the serum. From the properties of these fractions and of cobalt-serum, it seems that the cation is bound by the protein molecules at several different sites, which have different affinities for $Co^{2+}$. The possible significance of the greater loss of $Co^{2+}$ from cobalt-serum on dialysis against 0·154 M NaCl than against water as an indication of binding at carboxyl groups has been mentioned in the " Results " section, and in this connection it is known that ionic $Co^{2+}$ is bound by bovine serum albumin essentially at the free carboxyl groups of the protein (Lal and Rao, 1957). Binding at 2 additional sites is indicated by the fact that after exhaustive dialysis against NaCl solutions, some, but not all, of the residual $Co^{2+}$ is removed by further dialysis against EDTA at pH 7·5. One of these sites may be the imidazole ring (e.g. Lal, 1959) since about 13% of the bound $Co^{2+}$ of dialysed cobalt-serum becomes diffusible on photo-oxidation, a process that preferentially destroys histidyl residues of the proteins (Weil and Buchert, 1951; Weil and Siebler, 1953).

The partial removal of $Co^{2+}$ from dialysed cobalt serum by EDTA suggests the possibility of redistribution of the cation in the presence of an additional complexing agent, for example, another protein. Transfer of $Co^{2+}$ in this way might explain the inhibitory effect of cobalt-serum on pyruvate oxidation by isolated mitochondria. Alternatively, it is possible that the cobalt-serum complex is degraded rapidly in the mitochondrial system, since conventional methods for the isolation of these particles invariably yield preparations that are heavily contaminated with lysosomes. As demonstrated by the present experiments, digestion of cobalt-serum with a purified lysosomal proteinase liberates about 45% of the bound $Co^{2+}$ in a diffusible form, able to complex with Chelex resin. Although the bulk pH of the mitochondrial system is unsuitable for the activity of the lysosomal enzyme, protein adsorbed at the surface of the particles is likely to be subject to a more acid environment due to the transport of metabolically generated hydrogen ions.

Intracellular degradation of the cobalt-protein complexes by lysosomal proteinases probably contributes to the biological activity of cobalt-serum, and it seems significant that the separated cobalt-albumin fraction (Peak III, Fig. 4), which is particularly effective on the cell culture system, is more susceptible to enzymic hydrolysis than the 2 cobalt-globulin fractions (Table I). The effects of cobalt serum on cultured cells are very different from those of ionic $Co^{2+}$, and with the complex it has been possible for the first time to produce in myoblasts *in vitro* cytological changes that are similar to those of the early stages of tumour-induction *in vivo*. It is assumed that *in vitro* the cobalt-serum complex, possibly adsorbed at the surface of the myoblast, enters the cell by endocytosis. Subsequent digestion by lysosomal proteinase(s) leads to the liberation and redistribution of the bound $Co^{2+}$. A similar hypothesis has been proposed by Ryser and his colleagues (see Ryser, 1968, for a review) to explain the uptake and metabolism of $^{131}$I-labelled albumin and of a $Cd^{2+}$-ferritin complex by sarcoma S 180 II cells in culture. Eybl and Ryser (1964) conclude for example that the cellular damage produced by the $Cd^{2+}$-ferritin is due to the penetration of the complex into the cells and its subsequent degradation with the release of free $Cd^{2+}$ ions.

*In vivo* the formation of metal-protein complexes, analogous to cobalt-serum, at the site of implantation of the carcinogenic metals might provide carriers for the transport of the metallic ions into free myoblasts of the regenerating muscle and subsequently, into the cells of the developing rhabdomyosarcomata. The observed common pattern of intracellular distribution of $Co^{2+}$, $Ni^{2+}$ and $Cd^{2+}$ in the primary tumours induced by cobalt, nickel and cadmium (Heath and Webb, 1967) might follow from the redistribution of the cations on lysosomal degradation of the carrier proteins. In this connection it is possible that the " compounds " that are formed in the blood of rats on intravenous injection of $Be^{2+}$, and which are taken up by lysosomes of the liver (Witschi and Aldridge, 1968) might be complexes of the cation with proteins instead of, or in addition to, the postulated colloidal hydroxides and phosphates.

### SUMMARY

During the course of induction of rhabdomyosarcomata by implanted powdered metallic cobalt in rat skeletal muscle, the metal slowly dissolves and disappears from the injection site. In attempts to find a model for the dissolution process, cobalt metal powder has been incubated aseptically with horse serum at 37° C. Under these conditions, and with air as the gas phase, the metal dissolves slowly with the formation of complexes of both small molecular components and the proteins of the serum, binding by albumin being greater than by the globulins. In these proteins, $Co^{2+}$ ions appear to be bound at multiple sites, 2 of which may be free carboxyl groups and histidyl residues.

Cobalt-serum, produced by incubation of the metal with horse serum for 28 days, is not only less toxic for rat myoblasts in culture than the equivalent amount of ionic $Co^{2+}$ (as $CoCl_2$), but also produces cytological changes in the myoblasts that are similar to those seen in the vicinity of the implants of metal powder *in vivo*.

It is suggested that the cobalt-serum complex, possibly adsorbed at the surface of the myoblast, enters the cell by endocytosis. Subsequent digestion of the carrier-proteins by lysosomal proteinase(s) leads to the liberation and redistribution of the $Co^{2+}$ ions. In support of this hypothesis, a partially purified preparation of a lysosomal proteinase has been shown to degrade the cobalt-serum complex, and to liberate $Co^{2+}$ in a form that is freely-diffusible and which is retained by Chelex resin.

This work was done with the support of the British Empire Cancer Campaign for Research (J. C. Heath) and the Medical Research Council (M. Webb and M. Caffrey).

The authors are grateful to Miss A. Orledge for her skilled technical assistance.

### REFERENCES

AOKI, K., HARI, J. AND KAWASHIMA, K.—(1967) *Archs Biochem. Biophys.*, **120**, 255.
BARRETT, A. J.—(1967) *Biochem. J.*, **104**, 601.
DAVIS, B. J.—(1964) *Ann. N.Y. Acad. Sci.*, **121**, 404.
DINGLE, J. T., HEATH, J. C., WEBB, M. AND DANIEL, M. R.—(1962) *Biochem. biophys. Acta*, **65**, 34.
EYBL, V. AND RYSER, H. J.-P.—(1964) *Naunyn-Schmiedebergs Arch. exp. Path. Pharmak.*, **248**, 153.
FINDLAYSON, J. S.—(1965) *J. clin. Invest.*, **44**, 1561.
FLODIN, P.—(1962) ' Dextran gels and their applications in gel filtration '. Uppsala, Sweden (Pharmacia).
GILBERT, J. B., OTEY, M. C. AND PRICE, V. E.—(1951) *J. biol. Chem.*, **190**, 377.
HEATH, J. C.—(1956) *Br. J. Cancer*, **10**, 668.—(1960) *Br. J. Cancer*, **14**, 478.
HEATH, J. C. AND WEBB, M.—(1967) *Br. J. Cancer*, **21**, 768.
KOSZALKA, T. R. AND MILLER, L. L.—(1960) *J. biol. Chem.*, **235**, 663.
LAL, H.—(1959) *J. Am. chem. Soc.*, **81**, 844.
LAL, H. AND RAO, M. S. N.—(1957) *J. Am. chem. Soc.*, **79**, 3050.
LOWRY, O. H., ROSEBROUGH, N. J., FARR, A. L. AND RANDALL, R. J. (1951) *J. biol. Chem.*, **193**, 265.
PEDERSON, K. O.—(1962) *Archs Biochem. Biophys.*, Suppl. 1, 157.
RYSER, H. J.-P.—(1968) *Science, N.Y.*, **159**, 390.
SCHNEIDER, W. C.—(1948) *J. biol. Chem.*, **176**, 259.
SMITH, E. L.—(1948*a*) *J. biol. Chem.*, **173**, 571.—(1948*b*) *J. biol. Chem.*, **176**, 21.
TODD, S. M.—(1965) *Br. J. Cancer*, **19**, 444.
TOMMEL, D. K. J., VLIEGENTHART, J. F. G., PENDERS, T. J. AND ARENS, J. F.—(1968) *Biochem. J.*, **107**, 335.
WEIL, L. AND BUCHERT, A. R.—(1951) *Archs Biochem. Biophys.*, **34**, 1.
WEIL, L. AND SEIBLER, T. S.—(1953) *Archs Biochem. Biophys.*, **54**, 365.
WITSCHI, H. P. AND ALDRIDGE, W. N.—(1968) *Biochem. J.*, **106**, 811.
WOOD, E. J. AND BANISTER, W. H.—(1967) *Biochem. J.*, **104**, 42P.


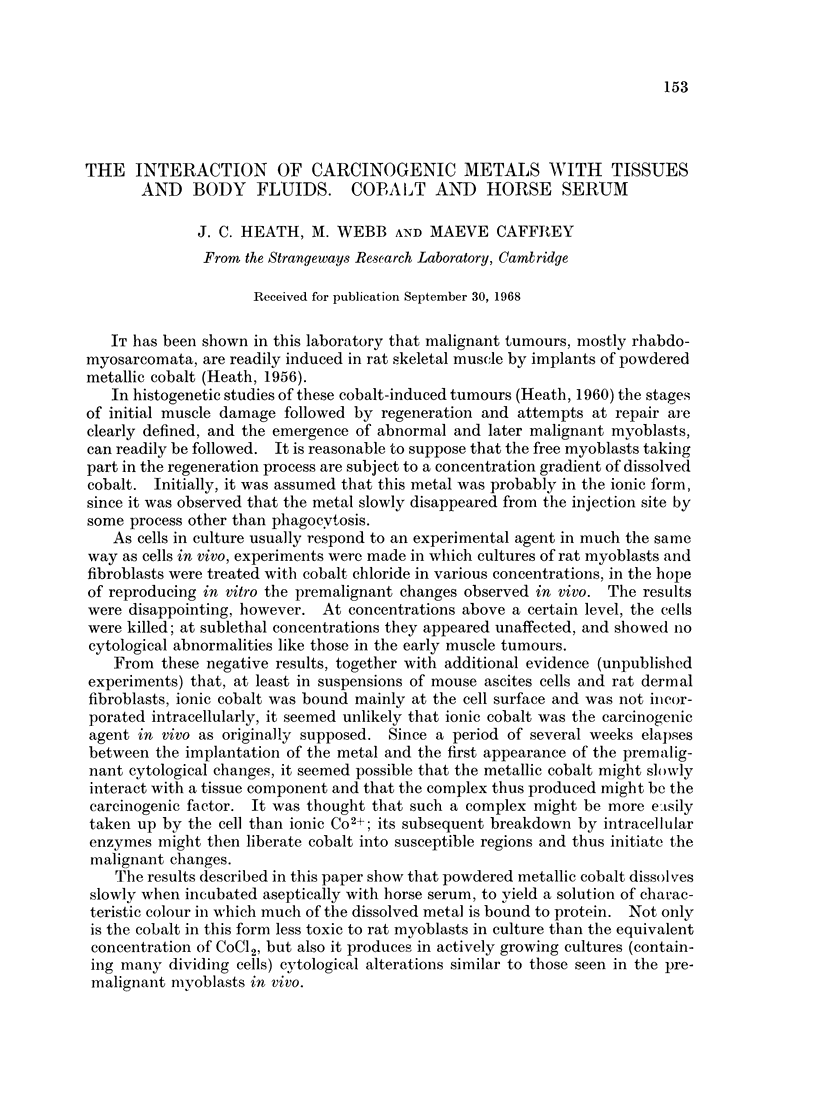

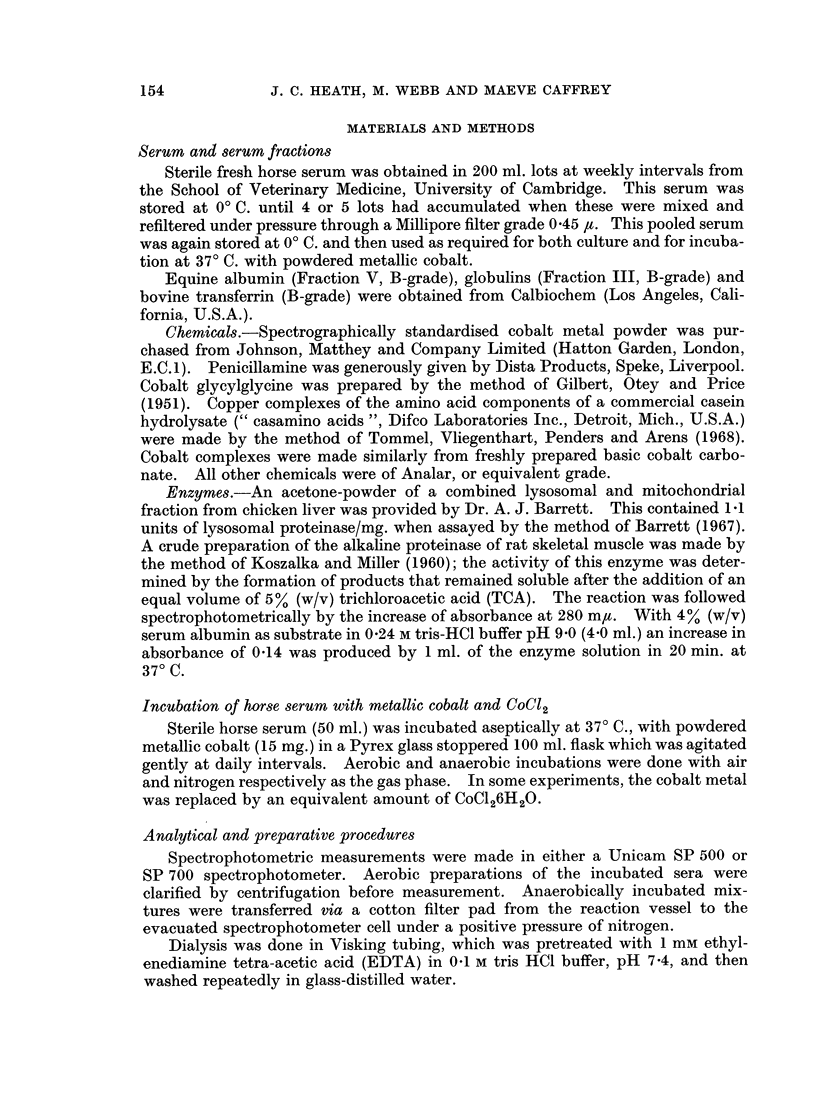

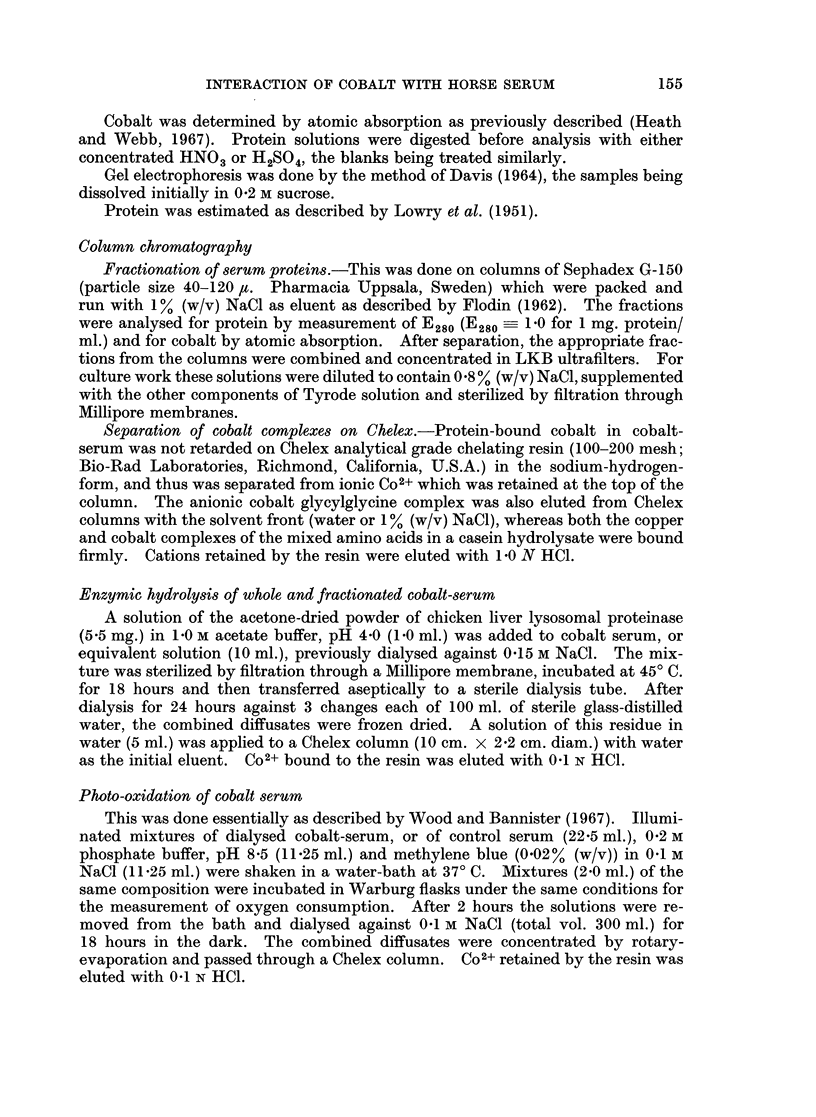

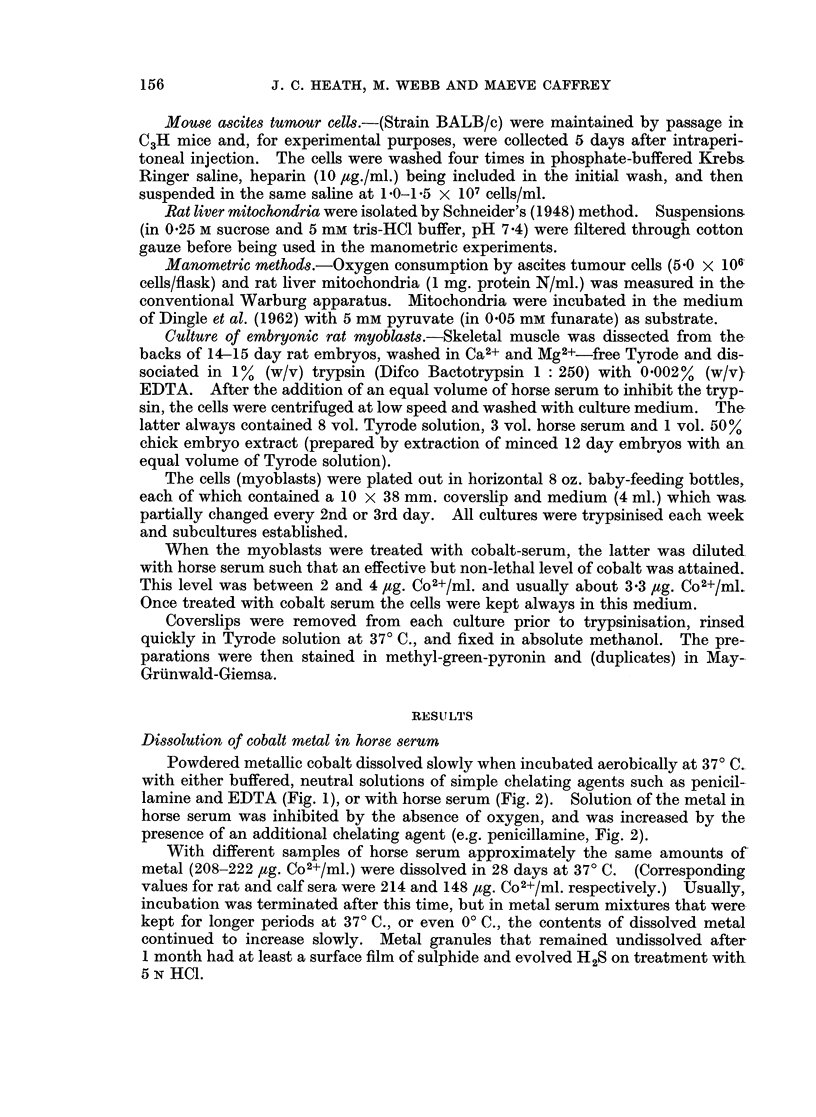

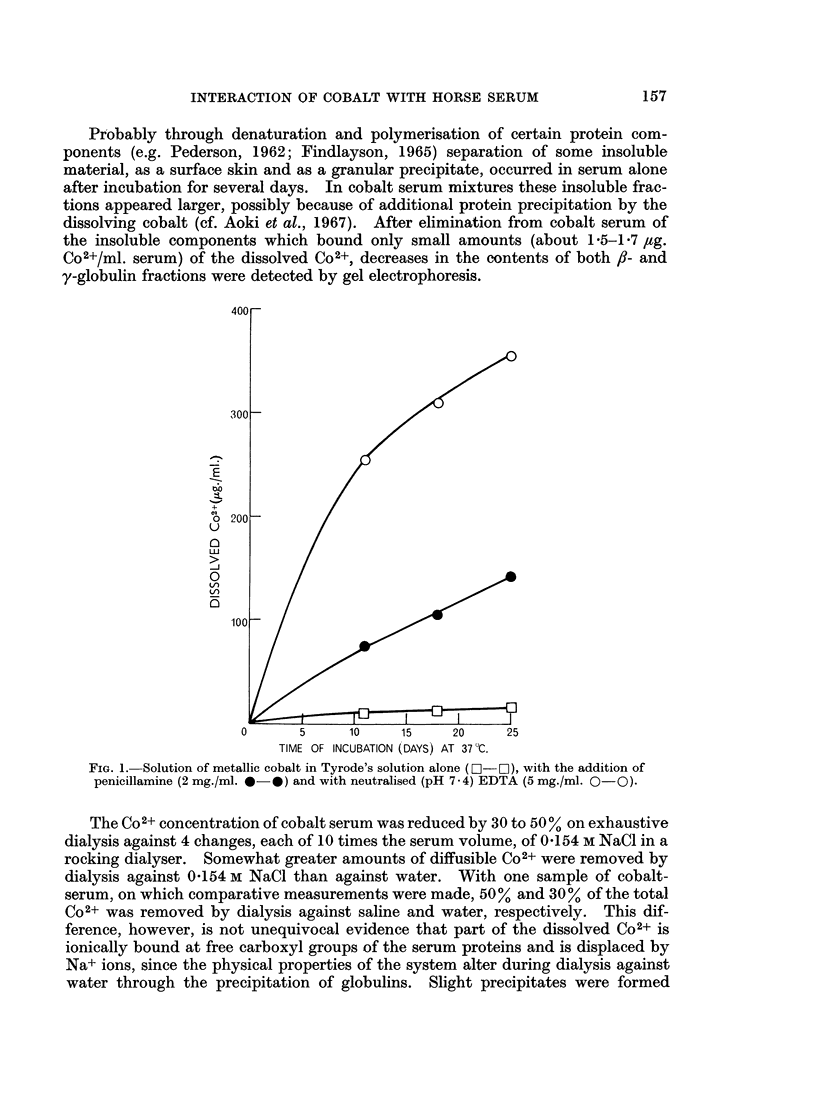

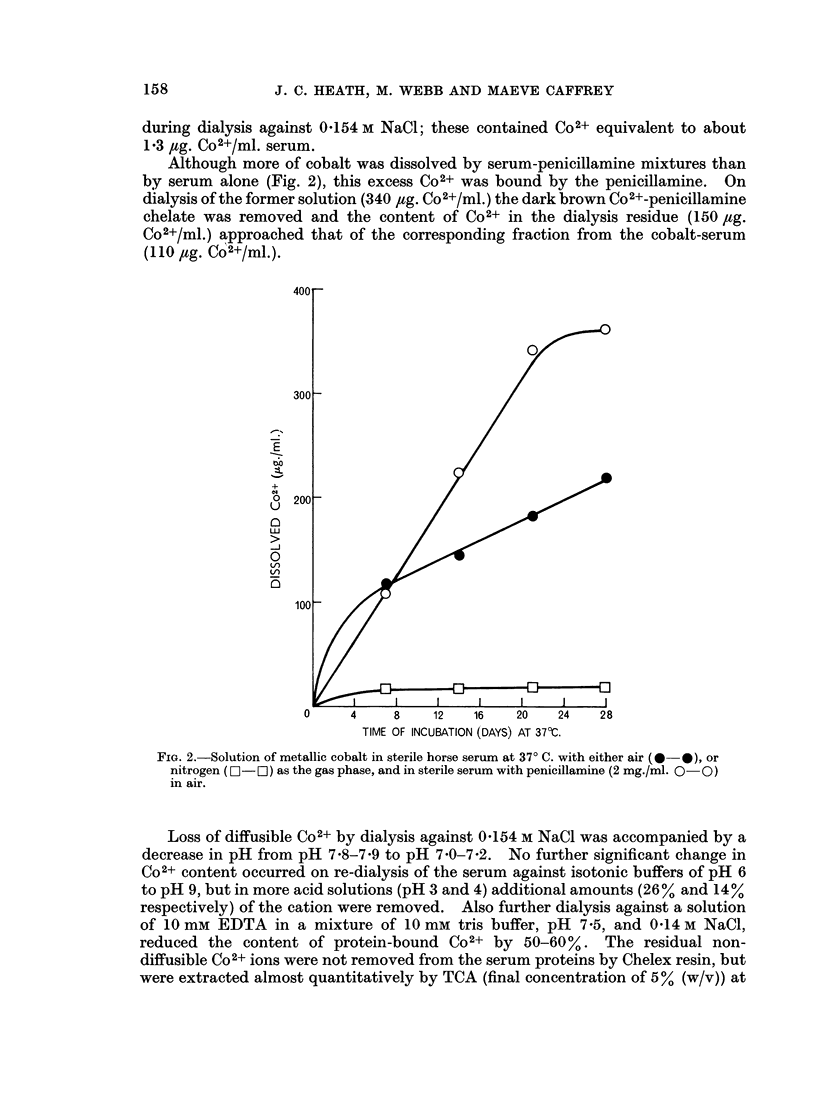

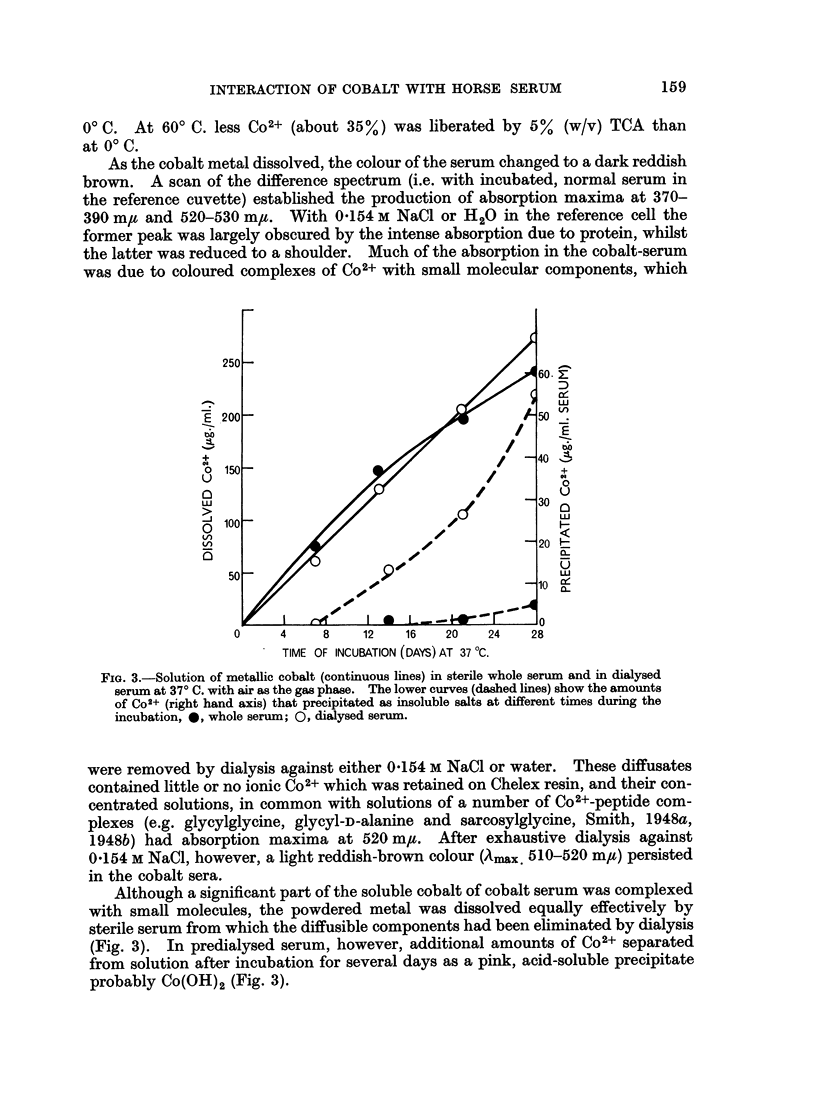

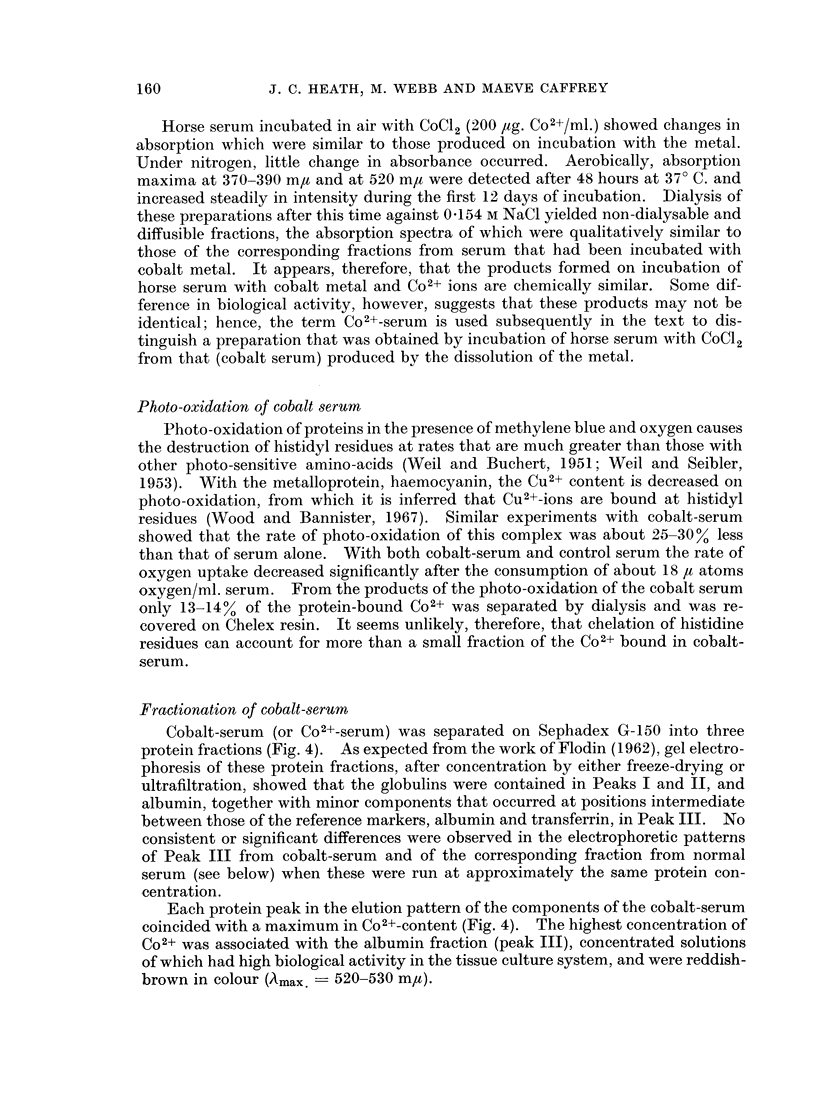

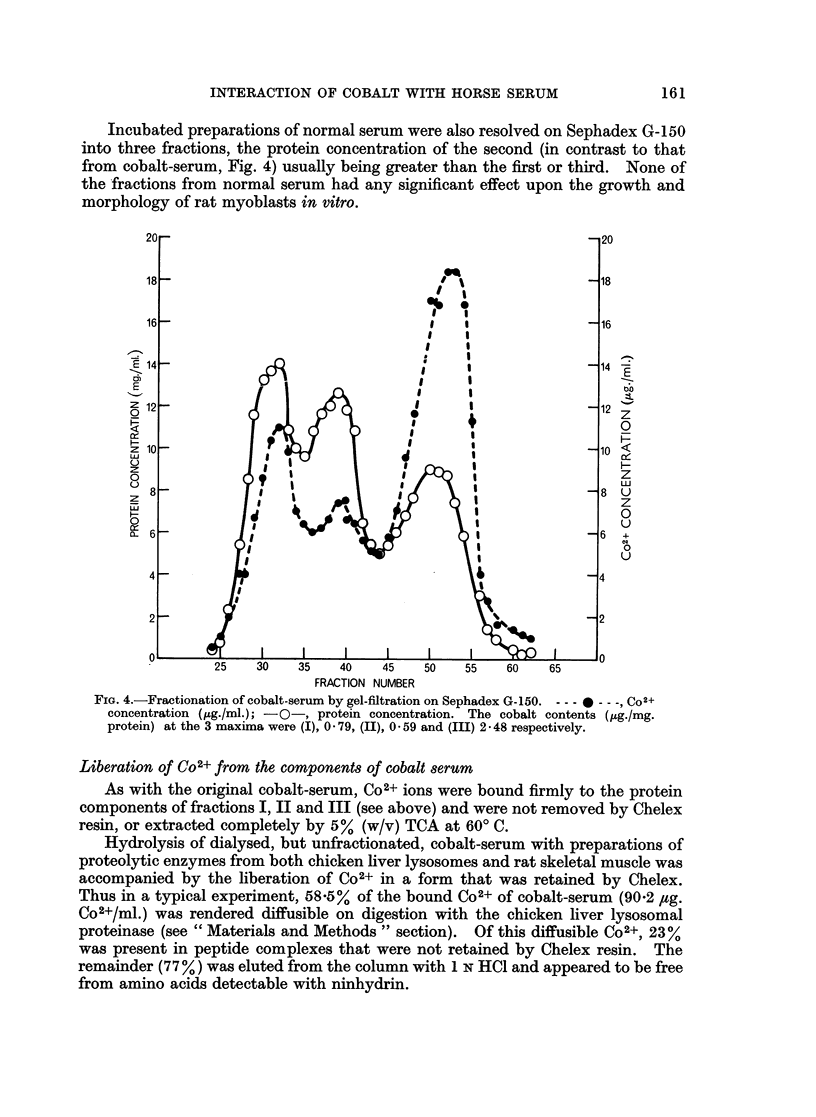

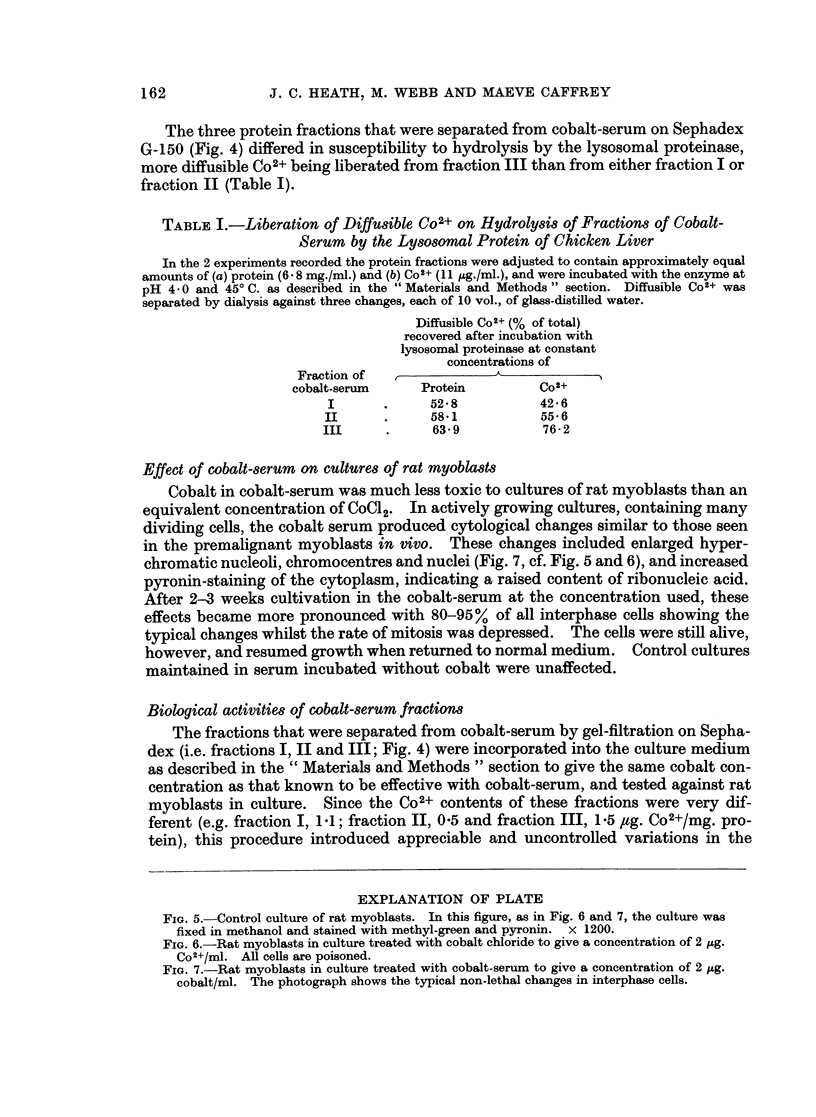

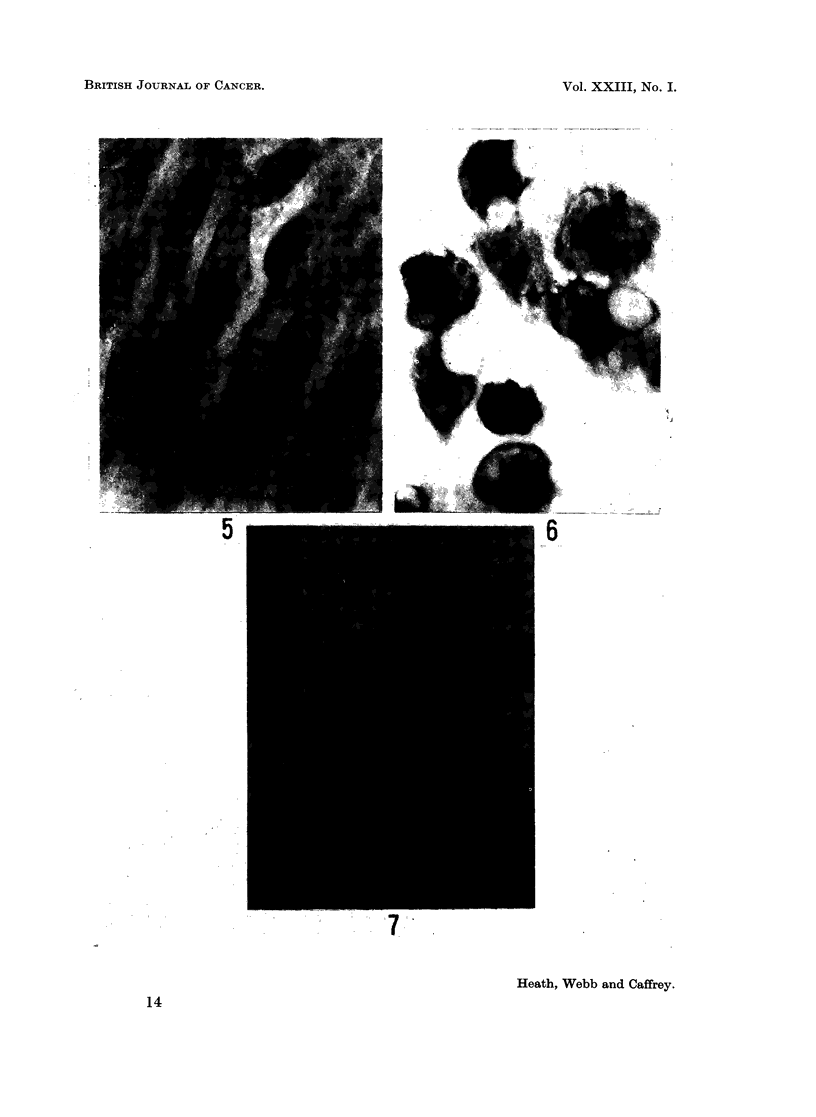

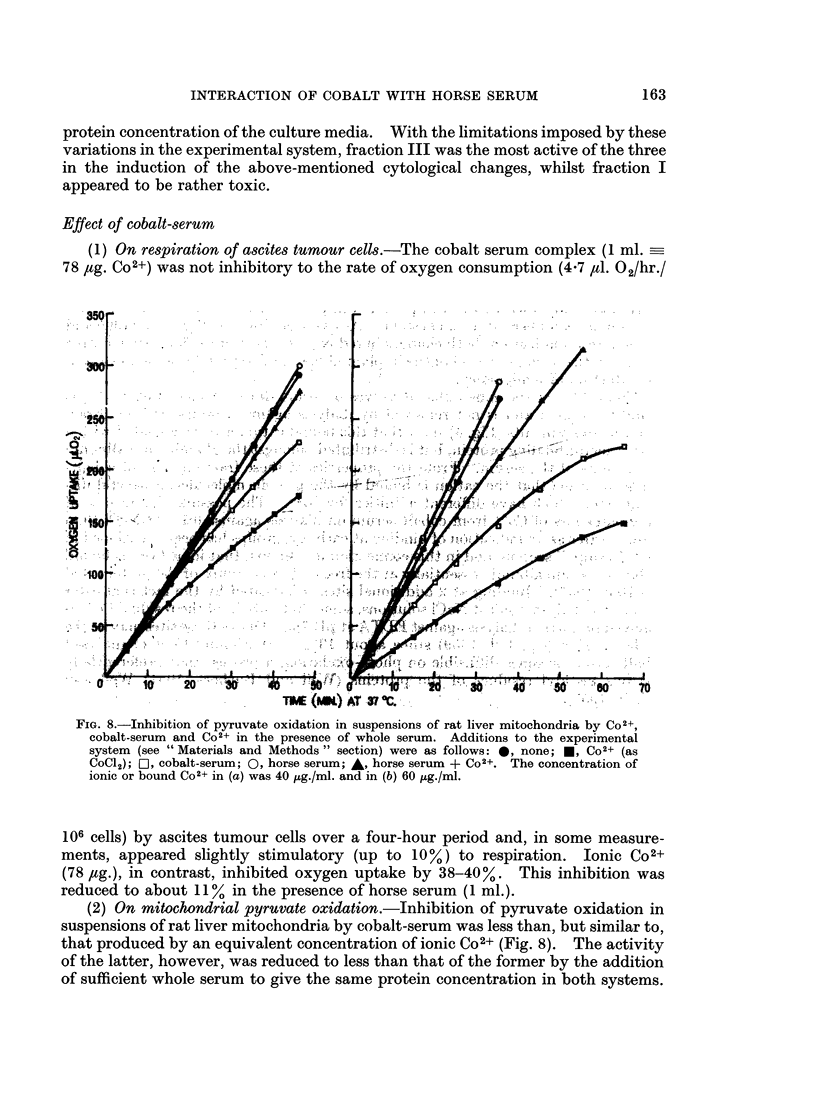

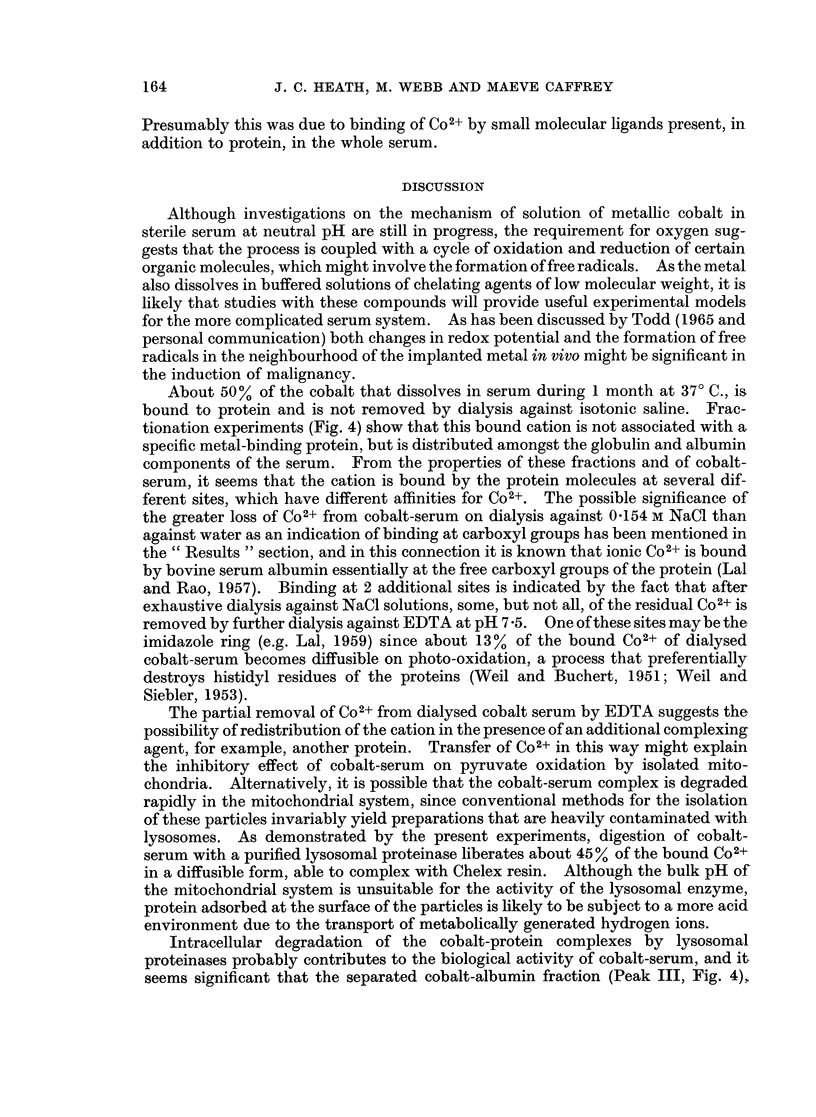

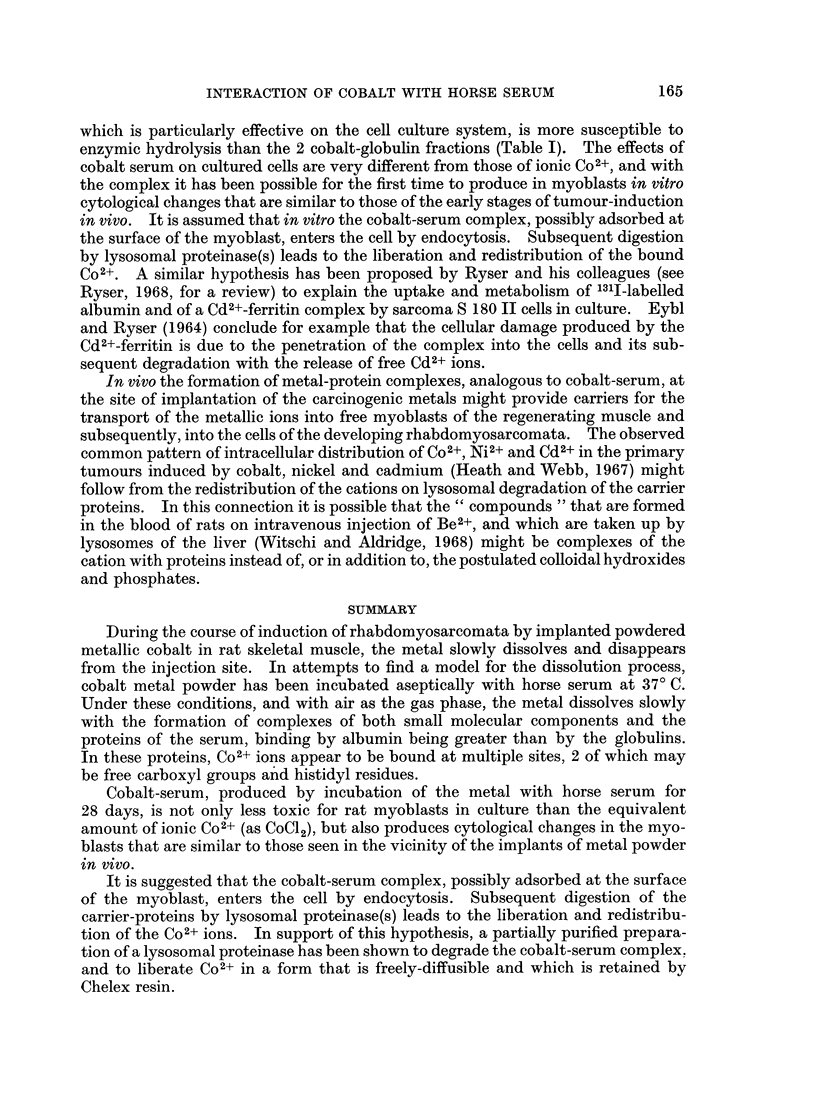

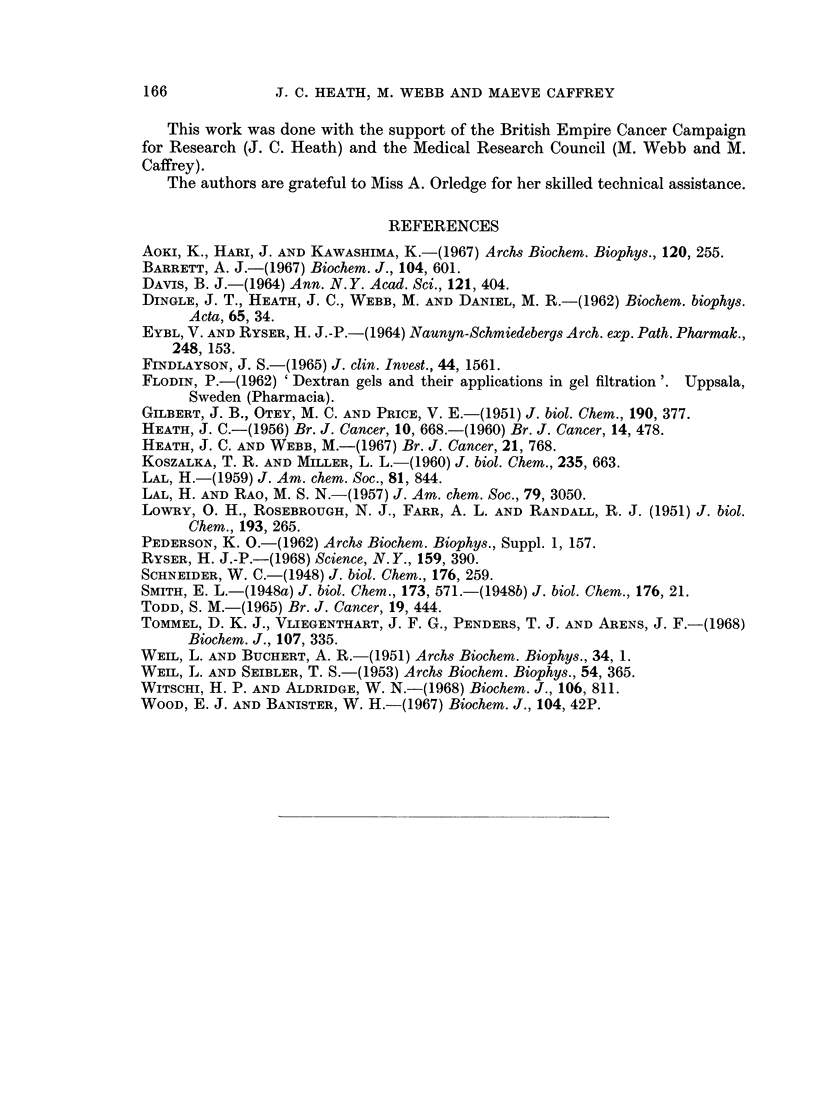


## References

[OCR_00759] Barrett A. J. (1967). Lysosomal acid proteinase of rabbit liver.. Biochem J.

[OCR_00761] DAVIS B. J. (1964). DISC ELECTROPHORESIS. II. METHOD AND APPLICATION TO HUMAN SERUM PROTEINS.. Ann N Y Acad Sci.

[OCR_00769] EYBL V., RYSER H. (1964). THE ACUTE TOXICITY OF CRYSTALLIZED FERRITIN, CADMIUM-FREE FERRITIN AND CDCL2 ON EHRLICH ASCITES CARCINOMA CELLS.. Naunyn Schmiedebergs Arch Exp Pathol Pharmakol.

[OCR_00777] GILBERT J. B., OTEY M. C., PRICE V. E. (1951). The enzymatic susceptibility of the red cobalt complexes of several dipeptides.. J Biol Chem.

[OCR_00778] Heath J. C., Webb M. (1967). Content and intracellular distribution of the inducing metal in the primary rhabdomyosarcomata induced in the rat by cobalt, nickel and cadmium.. Br J Cancer.

[OCR_00786] LOWRY O. H., ROSEBROUGH N. J., FARR A. L., RANDALL R. J. (1951). Protein measurement with the Folin phenol reagent.. J Biol Chem.

[OCR_00793] Ryser H. J. (1968). Uptake of protein by mammalian cells: an underdeveloped area. The penetration of foreign proteins into mammalian cells can be measured and their functions explored.. Science.

[OCR_00798] Tommel D. K., Vliegenthart J. F., Penders T. J., Arens J. F. (1968). A method for the separation of peptides and alpha-amino acids.. Biochem J.

[OCR_00805] Witschi H. P., Aldridge W. N. (1968). Uptake, distribution and binding of beryllium to organelles of the rat liver cell.. Biochem J.

